# Supplementary figures and images for: PRR14 overexpression promotes cell growth, epithelial to mesenchymal transition and metastasis of colon cancer via the AKT pathway
Source: PLoS One. 2019 Oct 9;14(10):e0218839. doi: 10.1371/journal.pone.0218839 (PMC6785111; doi:10.1371/journal.pone.0218839)

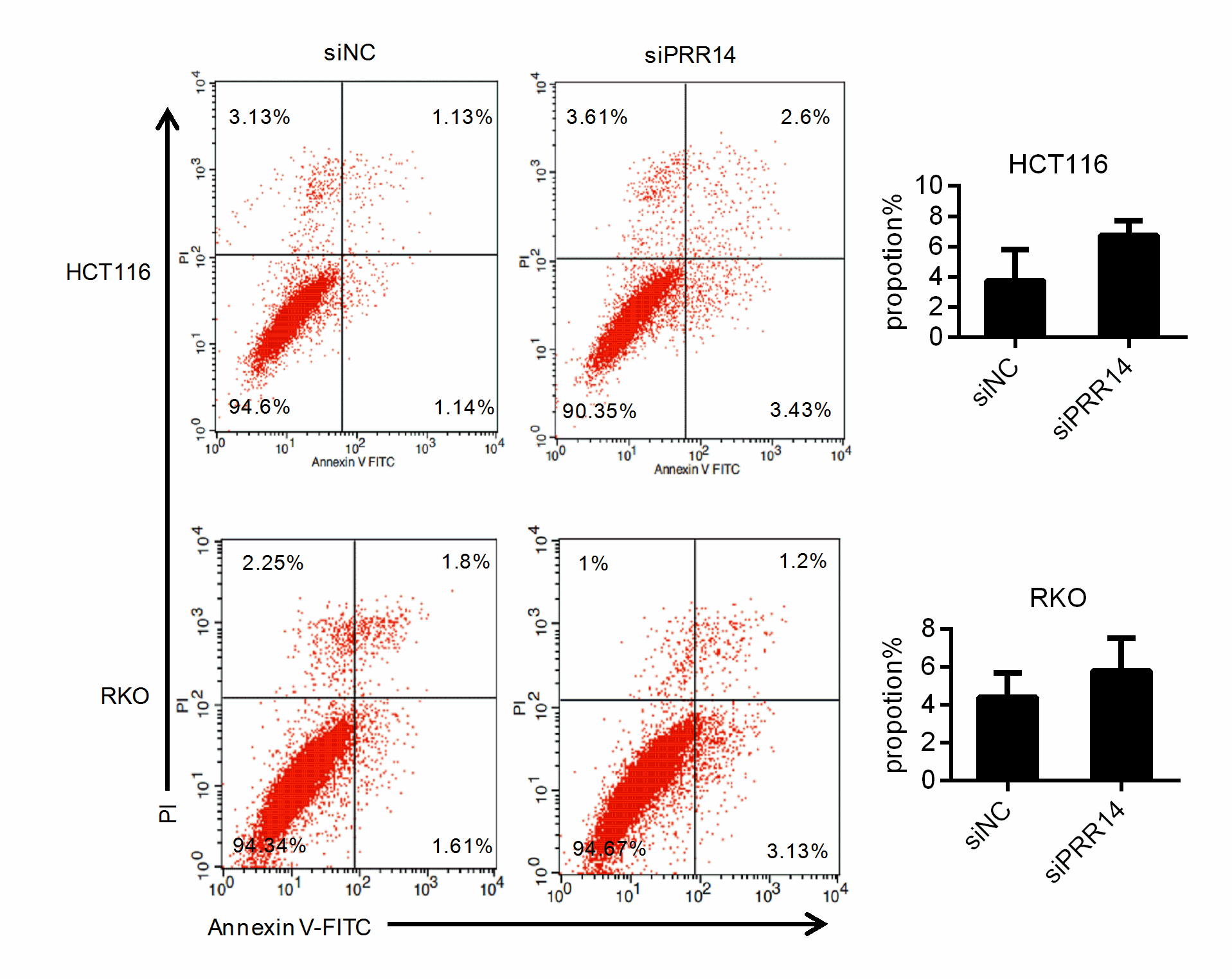

Supplement: S1 Fig — Representative images of apoptosis detection, and the proportion of the sum of early apoptosis and late apoptosis. (TIF) [file pone.0218839.s001.tif]
