# Supplementary material for: PRR14 overexpression promotes cell growth, epithelial to mesenchymal transition and metastasis of colon cancer via the AKT pathway
Source: PLoS One. 2019 Oct 9;14(10):e0218839. doi: 10.1371/journal.pone.0218839 (PMC6785111; doi:10.1371/journal.pone.0218839)
Supplement: S1 Table — (DOC) [file pone.0218839.s002.doc]

| Gene name | Primer sequence | Product length |
| --- | --- | --- |
| PRR14 | Forward:5’-GCACCACAGCTACCATCAGG-3’  Reverse:5’-CCGGTCCACCTTTTGTGAAG-3’ | 211bp |
| CDK1 | Forward:5’-AAACTACAGGTCAAGTGGTAGCC-3’  Reverse:5’-TCCTGCATAAGCACATCCTGA-3’ | 148bp |
| CDK2 | Forward:5’-CCAGGAGTTACTTCTATGCCTGA-3’  Reverse:5’-TTCATCCAGGGGAGGTACAAC-3’ | 90bp |
| CDK6 | Forward:5’-GCTGACCAGCAGTACGAATG-3’  Reverse:5’-GCACACATCAAACAACCTGACC-3’ | 225bp |
| CCNA | Forward:5’-CGCTGGCGGTACTGAAGTC-3’  Reverse:5’-GAGGAACGGTGACATGCTCAT-3’ | 120bp |
| CCNB | Forward:5’-AATAAGGCGAAGATCAACATGGC-3’  Reverse:5’-TTTGTTACCAATGTCCCCAAGAG-3’ | 111bp |
| CCND1 | Forward:5’-GCTGCGAAGTGGAAACCATC-3’  Reverse:5’-CCTCCTTCTGCACACATTTGAA-3’ | 135bp |
| CCNE | Forward:5’-GCCAGCCTTGGGACAATAATG-3’  Reverse:5’-CTTGCACGTTGAGTTTGGGT-3’ | 104bp |
| P21 | Forward:5’-TGTCCGTCAGAACCCATGC-3’  Reverse:5’-AAAGTCGAAGTTCCATCGCTC-3’ | 139bp |
| P27 | Forward:5’-AACGTGCGAGTGTCTAACGG -3’  Reverse:5’-CCCTCTAGGGGTTTGTGATTCT-3’ | 209bp |
| GAPDH | Forward:5’-GGAGCGAGATCCCTCCAAAAT-3’  Reverse:5’-GGCTGTTGTCATACTTCTCATGG-3’ | 197bp |
