# Supplementary material for: PRR14 overexpression promotes cell growth, epithelial to mesenchymal transition and metastasis of colon cancer via the AKT pathway
Source: PLoS One. 2019 Oct 9;14(10):e0218839. doi: 10.1371/journal.pone.0218839 (PMC6785111; doi:10.1371/journal.pone.0218839)
Supplement: Attachment — Submitted filename: Response to Reviewers.pdf [file pone.0218839.s003.pdf]

重慶醫科大學
CHONGQING MEDICAL UNIVERSITY

Department of Traditional Chinese Medicine,
Chongqing Medical University

No 1. Yixueyuan Rd. , Yuzhong District,
Chongqing, 400016 P.R.China
Phone: (0086) 02368485000
Fax: (0086) 02368485111
E-mail:fulijuan@cqmu.edu.cn

July 25, 2019

Dr. Jung Weon Lee
Editor
PloS One

Re: MS ID#: PONE-D-19-15816

Response to EDITOR

1. We would like to appreciate your careful consideration and the opportunity of resubmission. We have revised the manuscript as per your and reviewers' comments and construction suggestions. We have also tried our best to accomplish the editorial and experimental works so that the revised manuscript could meet the publication requirements from the journal.

2. Since the protocols we used are very general ones, we did not deposit our laboratory protocols in protocols.io. But we definitely could provide if required.

3. We also included our rebuttal letter (Response to Reviewers), marked-up copy (Revised Manuscript with Track Changes) and unmarked revised paper without tracked changes (Manuscript) in the submission system.

REVIEWER 1

We are very grateful to this reviewer's comments. Our responses to his/her comments are addressed below.

1. What is cellular localization of PRR14 in HCT/RKO cells? If predominantly nuclear, difficult to explain results. IHC in Fig. 1E is difficult to determine localization (higher mag as insert would help).

Response: We appreciate your kind reminder for checking PRR14 cellular localization. We have

detected it and added a set of images into the manuscript (Figure 2C).Please kindly find the revision in:
Section: Results – Knockdown of PRR14 inhibited cell growth in vitro and in vivo.
Pages: 12
Lines:238
Figure:2
The results showed that PRR14 staining was located in the nucleus, nuclear membrane and a small amount in the cytoplasm. Immunofluorescence staining of PRR14 protein has more power in localizing the PRR14 protein cellular level. This result is consistent well with the Proteinatlas database, https://www.proteinatlas.org/ENSG00000156858-PRR14/tissue/colon#img

We also inserted magnified figures of PRR14 staining in Figure 1E. It showed that PRR14's localization is mainly in the nucleus. However, we thought the intensive IHC staining of PRR14 in nuclear membrane might cover up the hematoxylin staining in nucleus. Hence, IHC could semi-quantify the PRR14 protein level in the tissue but not cellular level in this study.

2. What is effect of PRR14 knockdown on other signaling pathways such as ERK or JNK. Does PRR14 have a global role in intracellular signaling?

Response: As per your kind suggestions, We examined the effect of PRR14 knockdown on ERK and JNK pathways. Western blot results and semi-quantitative analysis of the histogram showed that no significant differences in phosphorylated and total protein levels of ERK and JNK were found in knockdown and controls cells. The figures and histogram were attached below. Our results indicate that PRR14 might not has close relation with ERK and JNK signaling pathway in HCT116 and RKO cell lines. Therefore, we did not put these results in revised manuscript.

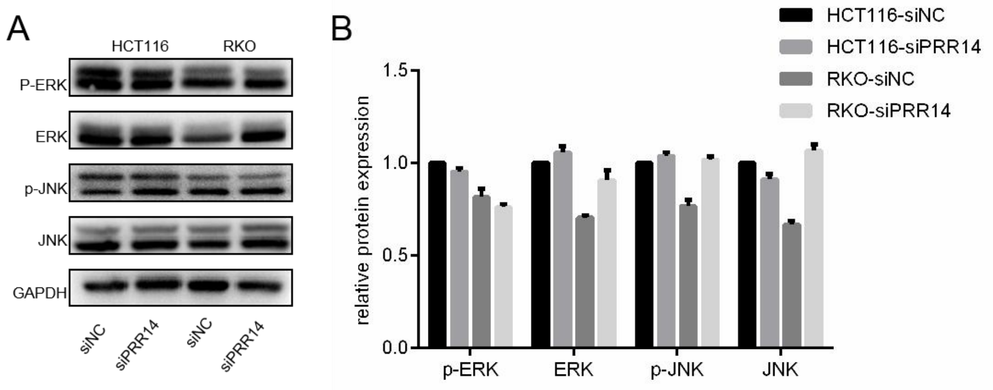

Figure legend: (A) Western blot was used to detect the ERK and JNK pathway genes expression in HCT116 and RKO cells transfected with siNC and siPRR14 siRNA. (B) Western blot results were analyzed statstically.

3. What is the cell cycle profile with PRR14 (G1/S block?).

Response: As per the reviewer's comments and suggestions, we additionally examined the effect of PRR14 expression on cell cycle progression, and the results were put in Figure 3. The results showed that PRR14 knockdown led to G1 phase arrest. Brdu labeling assay showed significant decline in percentage of S phase. Please kindly find the revision in
Section: Methods –Cell cycle and apoptosis analysis, immunofluorescent assay

Pages:8 and 9
Paragraph: 1 and 5
Lines: 145 and 183

Section: Results – Knockdown of PRR14 resulted in cell cycle arrest at G1 phase
Pages: 13
Paragraph: 4
Lines:257
Figure:3

4. Further comment on differences in PRR14 kd in 2 cell lines. E.g., p21/p27 mRNA; despite no change p27 mRNA, protein increased in RKO.

Response: We appreciate your critical comments on inconsistent expression of P21 and P27 in PRR14 kd in 2 cell lines. We think that the inconsistent mRNA expression of P21 and P27 after PRR14 knockdown indicates that the mechanism of action may be inconsistent between the two cells in mRNA level. But we do not know why and feel sorry for not studying it further since its protein level changes may make sense. The western blot results showed that the expression of P21 and P27 increased after PRR14 knockdown in both cell lines. We speculate that PRR14 plays a more important role in the post-translation level. We reinterpreted this in the paper. Please kindly find the revision in
Section: Results – Knockdown of PRR14 affected the expression of cell cycle-related genes and AKT pathway genes.
Pages: 16
Paragraph: 3
Lines:311

Section: Discussion
Pages: 17
Paragraph: 3
Lines:339

5. Table 2 distant mets n=4 all with high PRR14 (100%), whereas low n=0 yet number in parenthesis is 48.8%; should be 0. How explain P=0.029 for TNM but 0.079 ln mets?

Response: we appreciate the reviewer's meticulous evaluation. We apologize for this mistake.The original Table 2 has been changed to Table 1, The correct number in Table 1 parenthesis is n=0. We have corrected it accordingly. Please kindly find the revision in
Section: Results – PRR14 is highly expressed in colon cancer, and this correlates with poor prognosis of the disease.
Pages: 11
Paragraph:3
Lines:228
Table:1
Group: Distant metastasis-PRR14^{low}

As for the *P* value, in TNM staging system, T,N,M represents three indicators respectively: primary tumor,

regional lymph node involvement and distant metastasis. TNM staging is a combination of the three indicators. Therefore, the two values may be inconsistent.

6. Tumor xenografts---presumably kd is transient if using siRNA. Level of PRR14 in tumors? How long after siRNA transfection does PRR14 kd last in vitro?

Response: We greatly appreciate your critical comment in strengthening this study. We tested the interference effect of HCT116 cells on 2 to 10 days after transfection with siRNA, and the results were shown in below (Figure A). The Western blot results and semi-quantitative analysis showed that PRR14 expression was gradually increased during 10 days following knock down by siRNA, but still significantly lower than the control cells. We think the PRR14 expression would be comparable in siRNA kd cells and control cells if time went on.

Since siRNA could transiently knock down gene expression as mentioned by reviewer and others, and siRNA might not be such effective like shRNA. We were curious about our results, firstly, as per reviewer's kind suggestions, we then detected the expression of PRR14 in subcutaneous tumors by Western blot. We pooled tumors of two groups (No.9 and 10) since their small size. The results showed PRR14 expression was significantly lower in siRNA tumors than controls (Figure B). We postulated that although some cells restored their PRR14 expression in siRNA kd xenograft cells, their proliferation has been inhibited in the first 10 days following grafting. Therefore, the size in siRNA kd xenograft was smaller than controls. SiRNA has been also used in tumor xenografts in some of qualified studies which further convinced our results were correct. Here we listed several of them.
https://www.cell.com/molecular-therapy-family/nucleic-acids/pdf/S2162-2531(16)30300-6.pdf
https://www.ncbi.nlm.nih.gov/pubmed/23095742
https://www.nature.com/articles/s41598-017-07973-4

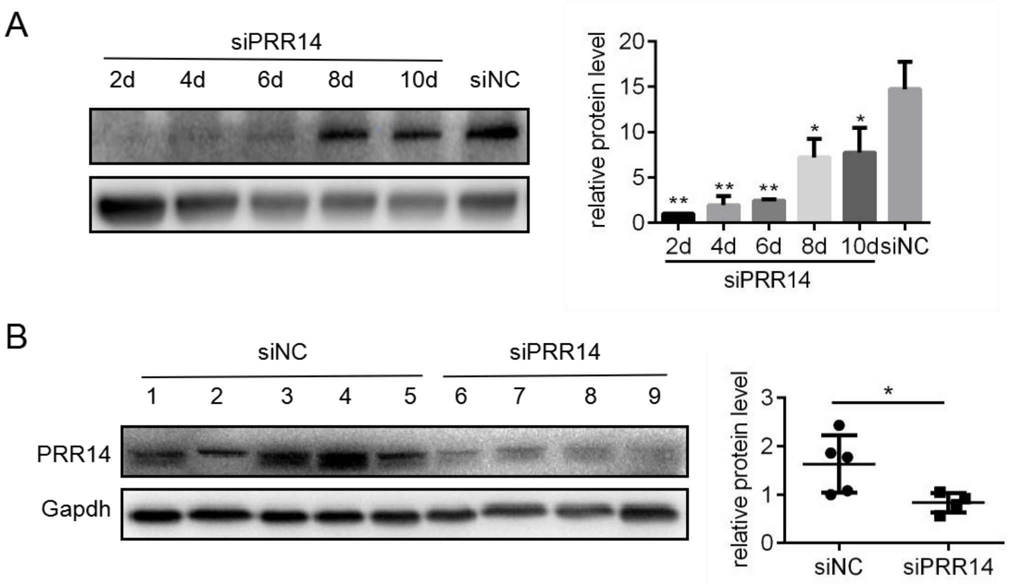

Figure: (A) Western blot was used to test the interference effect of HCT116 cells on 2 to 10 days after transfection with siRNA, and the results were statistically analyzed. (B) Western blot was used to detect the PRR14 expression in subcutaneous tumors, and the results were statistically analyzed. (*: $p < 0.05$; **: $p < 0.01$).

REVIEWER 2

1. Major comments
HCT116 and RKO mesenchymal one. What happened if PRR14 is overexpressed (or silenced) in epithelial colon cancer cells? Some keys experiments should be presented in epithelial colon cancer cells. On the same way it would be interest to see the effect of PRR14 overexpression in HCT116 and RKO cells. Also if the results are negative they should be presented.

Response: Indeed, it will be more convincing if we get an overexpression results in mesenchymal cells, we apologize for our insufficient consideration and planning. Unfortunate, we do not have the overexpression plasmids and cell line as above mentioned.

However, we compared EMT indicators expression in xenograft tumor cells of siRNA and control, and put them in Figure 5C and D. It found that mesenchymal phenotype markers-Twist1 and Vimentin were highly expressed in the control group, while epithelial phenotype marker Ecad was generally higher in the siRNA group. This results confirmed that PRR14 expression level was correlated with EMT phenotype.Please find them in
Section: Results – Knockdown of PRR14 affected the expression of EMT-related genes and the formation of pseudopodia.
Pages: 15
Paragraph:2
Lines: 287
Figure: 5C and D

2. Minor comments
The sentence “To clarify the mechanism by which PRR14 regulates the cell cycle” at the beginning of the result section “Knockdown of PRR14 affected the expression of cell cycle-related genes and AKT pathway genes” is not correct. Indeed, the authors did not investigate at all the ability of PRR14 to regulate cell cycle but they address the point of its role in proliferation (not in specific cell cycle phases).

Response: Based on the reviewer's comments, we examined the effect of PRR14 knockdown on cell cycle progression, and found that PRR14 knockdown resulted in G1 phase arrest. Brdu labeling assay showed a decreased cell proportion in S phase, we put them in Figure 3. This indicates that PRR14 plays an important role in cell cycle progression. Please kindly find the revision in
Section: Methods –Cell cycle and apoptosis analysis, immunofluorescent assay
Pages: 8 and 9
Paragraph: 1 and 5
Lines: 145 and 183

Section: Results – Knockdown of PRR14 resulted in cell cycle arrest at G1 phase
Pages: 13
Paragraph: 4
Lines:257
Figure:3

3. Figure 5C is not mentioned in the text

Response: We apologize for our carelessness, the original Figure 5C has been changed to Figure 6C, and we have added the interpretation and discussion of Figure 6C. Please kindly find the revision in Section: Results – Knockdown of PRR14 affected the expression of cell cycle-related genes and AKT pathway genes
Pages: 16
Paragraph: 3
Lines: 311

Section: Discussion
Pages: 17
Paragraph: 3
Lines:339

We again appreciate your time and efforts in handling this manuscript. We hope that the revised manuscript is satisfactory to you and reviewers for consideration of publication in PloS One.

Sincerely,

Lijuan Fu, Ph.D. Associate Professor
